# Methotrexate-Associated Lymphoproliferative Disorder Developed Ectopically in the Maxillary Gingiva and Bilateral Lungs

**DOI:** 10.1155/2020/4814519

**Published:** 2020-04-27

**Authors:** Kyoichi Obata, Tatsuo Okui, Koji Kishimoto, Soichiro Ibaragi, Akira Sasaki

**Affiliations:** ^1^Department of Oral and Maxillofacial Surgery, Okayama University Graduate School of Medicine, Dentistry and Pharmaceutical Sciences, Okayama, Japan; ^2^Department of Dentistry and Dental Surgery, Tsuyama Central Hospital, Okayama, Japan

## Abstract

A 58-year-old Japanese woman complained of a painful right maxillary premolar gingiva and ulcer. The patient had RA and had been treated with several immunosuppressive drugs such as methotrexate. Head and neck CT indicated no obvious bone destruction with maxillary. However, chest CT indicated the presence of nodular mass of the bilateral lungs. FDG-PET/CT indicated the presence of increased uptake in both lesions. On immunohistochemistry, atypical large-sized lymphocytes were positive for CD20 and EBER-ISH and negative for CD3, CD5, and CD10; the Ki-67 labeling index was high, the histopathological diagnosis was EBV-positive DLBCL, and the clinical diagnosis was MTX-LPD. The patient's treatment with MTX was then discontinued; we removed the alveolar bone which necrosed after 5 weeks. The lesion and the nodular mass at the bilateral lungs had completely disappeared after 7 weeks.

## 1. Introduction

Methotrexate (MTX) is an anchor drug used to treat rheumatoid arthritis (RA), and several cases of the occurrence of MTX-associated lymphoproliferative disorder (MTX-LPD) have been reported [1]. According to the World Health Organization (WHO) Classification, MTX-LPD is classified as an “other iatrogenic immunodeficiency-associated lymphoproliferative disorder” [2]. Of the reported cases of patients diagnosed with MTX-LPD, 40%–50% have been extranodal, occurring at sites of thoracicoabdominal organs. MTX-LPD occurs at sites in the oral cavity such as the gingiva, tongue, and mouth floor, but such cases are rare [2]. A case of a subtype of MTX-LPD, i.e., Epstein–Barr virus- (EBV-) positive mucocutaneous ulcer (EBV-MCU) was reported, but there have been very few comparisons of MTX-LPD and EBV-MCU in the oral region. Here, we report an extremely rare case of a patient with RA who developed MTX-LPD in the maxillary gingiva and bilateral lungs, and we compare EBV-MCU and MTX-LPD.

## 2. Case Report

The patient was a 58-year-old Japanese woman who visited the Department of Dentistry and Oral Surgery, Tsuyama Central Hospital (Okayama, Japan) at the end of May 2018, complaining of a painful right maxillary premolar gingiva and ulcer for the most recent 2 weeks. The patient had had RA for 10 years and had been treated with several immunosuppressive drugs, including MTX (2 mg/week for 6 months and 4 mg/week for 6 months) and prednisolone (PSL: 2 mg/week for 10 years).

**Figure 1:**
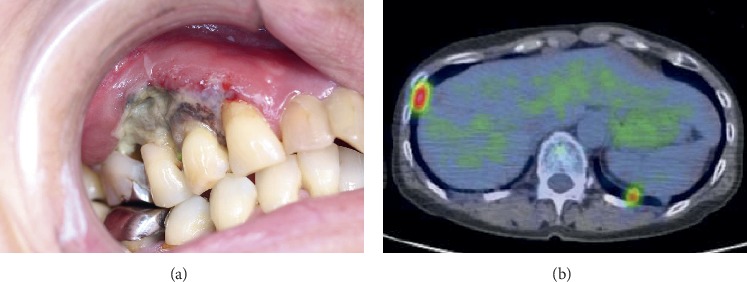
(a) Intraoral photograph: a painful necrotic ulcer in the right maxillary gingiva. (b) Horizontal FDG-PET/CT: pathological uptake of FDG at the bilateral lung.

On clinical examination, a right submandibular lymph node was swollen, with pain. The intraoral examination showed swelling with necrotic ulcer formation of the right maxillary gingiva in the canine to the first molar region. There was no exposure of perilesional alveolar bone. The perilesional premolar showed mild mobility with occlusal and percussion pain (Figure 1(a)). A blood examination revealed EBV infection (EBV viral capsid antigen IgG = 320, IgM < 10, IgA < 10, and EBV nuclear antigen = 80).

There was no obvious bone destruction with lesion anywhere as shown by dental radiographs and head and neck computed tomography (CT). Lymph node swellings were not detected except for the right submandibular lymph node. However, chest CT indicated the presence of a nodular mass that was suspected to be a malignant lung tumor of the bilateral lungs, and S8 of the inferior lobe of the right lung was the largest. Fluorodeoxyglucose-positron emission tomography/CT (FDG-PET/CT) indicated the presence of increased uptake in the right maxilla (maximum standardized uptake value: SUVmax = 5.85) and bilateral lungs (S8 of the inferior lobe of the right lung SUVmax = 4.94) but no uptake in the right submandibular lymph node (Figure 1(b)).

**Figure 2:**
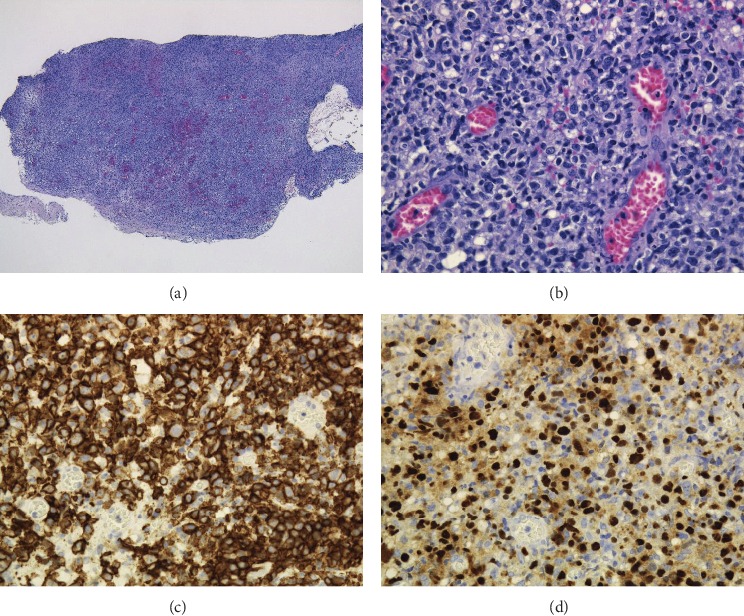
Histopathology results. The histopathological diagnosis was EBV-positive diffuse large B-cell lymphoma (DLBCL). (a) H&E stain (×40) showed large numbers of lymphocytes accumulation in the submucosal stroma. (b) H&E stain (×200): lymphocytes accumulation was atypical and large sized with a background of inflammatory cell infiltration. Immunohistochemistry stain (×200): tumor cells showed strong positivity for CD20 (c) and EBER-ISH (d).

We performed a biopsy based on a suspicion of MTX-LPD since the patient had been treated with MTX to treat RA for approximately one year. Hematoxylin and eosin staining showed a high degree of atypical large-sized lymphocyte infiltration with a background of inflammatory cell infiltration in the submucosal stroma. Immunohistochemistry staining showed that the atypical large-sized lymphocytes were positive for CD20 and negative for CD3, CD5, and CD10; the Ki-67 labeling index was high. The result of EBV-encoded small RNA in situ hybridization (EBER-ISH) was positive, and the histopathological diagnosis was EBV-positive diffuse large B-cell lymphoma (DLBCL) (Figure 2).

It was not possible to make a diagnosis of bilateral lung lesions because the patient refused an endoscopic biopsy, but a hematologist and respiratory medicine physician at another hospital indicated that the lung lesion was also MTX-LPD. From the results obtained in these examinations, we diagnosed MTX-LPD that had developed ectopically and was expressed at the same time in the maxillary gingiva and bilateral lungs.

We consulted the patient's primary physician, and the patient's treatment with MTX was then discontinued. Two weeks after the discontinuation of MTX, the ulcer and swelling in the gingival lesion were reduced, and the swelling and pain in the right submandibular lymph node had resolved.

The size of the nodular mass at the bilateral lungs also began to reduce. At 4 weeks after the discontinuation of MTX, we confirmed that the ulcer had disappeared completely, but the perilesional alveolar bone was exposed due to shedding of the necrotic gingiva. At 5 weeks, the exposed alveolar bone necrosed, and we removed sequestrum along with the premolar of the patient under local anesthesia.

At 2 weeks after this surgery, the wound was covered by normal epithelium, and the lesion had completely disappeared. The nodular mass at the bilateral lungs had also completely disappeared. The patient's RA had slightly worsened with joint pain, but her rheumatologist maintained good control of the symptoms with analgesics without a change to another drug for RA. At 15 months since the withdrawal of MTX, the patient was in a good condition without recurrence.

## 3. Discussion

In 1991, Ellman et al. first reported MTX-LPD as a type of malignant lymphoma that develops in patients with RA [3]. Lymphoproliferative disorders (LPDs) can develop in individuals with an autoimmune disease who are treated with immunosuppressive therapy. It was also reported that RA patients in particular are at an increased risk of developing LPDs due to their RA itself, regardless of the presence/absence of immunosuppressive therapy [4]. Accordingly, patients with an autoimmune disease that is being treated with immunosuppressive therapy are suspected to be at risk for LPDs, but it is less well understood how MTX-LPD is directly related to iatrogenic immunosuppression.

MTX-LPD has been reported to occur in various parts of the body, and recently the number of MTX-LPD cases in the oral cavity has increased. However, our review of the reported MTX-LPD cases leads us to note that the clinical features and pathological conditions differ significantly between the MTX-LPD that developed in the oral cavity and the MTX-LPD that developed at other body sites.  Table 1 summarizes the results of our review of the 51 reported cases of MTX-LPD in the oral cavity (including our patient's case) in comparison with the reported MTX-LPD cases at other body sites (*n* = 84) (Table 1).

The average age of the patients with MTX-LPD in the oral cavity is 70.2 years (range 40–87 years), and the male-to-female ratio is 1 : 2.64, revealing a tendency toward females. In addition, 98.0% of these patients were treated with a low-dose MTX for RA; the average period from the diagnosis of RA was 142.8 months (range 6–396 months), and the duration of MTX treatment was 99.0 months (range 1–360 months).

Compared to the cases of MTX-LPD at other body sites, there were almost no differences in age or the male-to-female ratio, but the period from the diagnosis of RA (37.3 months) and the duration of MTX treatment (42.3 months) were much longer in the cases of MTX-LPD in the oral cavity.

We next examined the ectopic cases of MTX-LPD corresponding to Stages 2–4 in the Ann Arbor classification, and we observed that the number of cases in the oral cavity was significantly lower than the number in the rest of the body (21.6% vs. 83.3%, *x*^2^(1) = 53.75, *p* < 0.01). The reoccurrence rate was also significantly lower in the oral cavity compared to the rest of the body (3.9% vs. 21.6%, *x*^2^(1) = 7.26, *p* < 0.01). Conversely, the EBV infection rate was significantly higher in the oral cavity than in the rest of the body (96.1% vs. 29.8%, *x*^2^(1) = 52.74, *p* < 0.01). Our comparison of these two patient groups thus revealed that regarding the occurrence of MTX-LPD, there are several important differences between the oral cavity and the rest of the body. To further investigate this issue, we also investigated whether EBV-MCU might be a subtype of MTX-LPD.

EBV-MCU is an ulcer in a cutaneous or mucosal site. In 2010, Dojcinov proposed that EBV-MCU occurs in patients with age-related or iatrogenic immunosuppression, and in 2016, EBV-MCU was newly added to the WHO Classification of Tumors of Haematopoietic and Lymphoid Tissues [5, 6]. EBV-MCU is defined as a B-cell lymphoproliferative disorder that is associated with defective surveillance for EBV and that frequently occurs in the oral mucosa, especially the gingiva; however, the pathogenesis of EBV-MCU is not yet known, in part because there have been so few reports [5, 7].

EBV infects >90% of world's population and usually continues to be suppressed by cell-mediated immunity after the primary infection. However, it is reported that EBV activation by some types of stimulation such as immunosuppression causes various diseases, including nasopharyngeal cancer. Certain immunosuppressants further promote EBV activation. MTX treatment was reported to activate the EBV immediate-early promoters and promote EBV replication [7–9].

EBV-MCU usually presents at a site that is subjected to many chronic stimuli (e.g., the oral cavity, skin, and gastrointestinal tract) [5]. The oral cavity has many chronic stimuli; examples include periodontal disease and ill-fitting dentures, which cause a local proliferation of EBV-infected B cells [10]. There are many EBVs in the saliva, gingival crevicular fluid, and gingival tissue of patients with periodontal disease. Imai et al. reported that the anaerobe Gram-negative periodontal bacterium *Phorphyromonas gingivalis* induces EBV activation [11]. The causes of EBV-MCU are still unknown; however, we speculate that chronic irritation owing to periodontal disease and poor denture stability are related with the occurrence of EBV-MCU in MTX-treated patients.

Age-related EBV-MCU due to immunosuppression is difficult to treat, but EBV-MCU due to immunosuppressive treatment can have a benign course with the withdrawal of an immunosuppressive drug such as MTX. Even if the lesion does not improve, chemotherapy works extremely well for EBV-MCU. It was reported that 96.6% of patients with EBV-MCU attain a complete remission, and there is no reported death due to this disease [12].

MTX-LPD reported in the oral cavity is thus highly likely to be EBV-MCU since the reported cases of MTX-LPD in the oral cavity showed a high local EBV infection rate, few ectopic cases, and good prognoses compared to MTX-LPD at other sites in the body. However, this is only our speculation, as there is no definitive evidence for this. In our patient's case, we diagnosed MTX-LPD because lesions also occurred in the lungs, but we also considered the possibility of EBV-MCU. EBV-MCU is a subtype of MTX-LPD, but there are still many points to consider before this disease entity is fully explained. A further accumulation of cases and analyses are necessary to establish the entire clinical picture and condition, including the histopathologic features of MTX-LPD and EBV-MCU.

## Figures and Tables

**Table 1:** Clinical findings of 51 cases of MTX-LPD in the oral cavity and 84 in the whole body.

	Oral cavity	Whole body
No. of cases	51	84
Age		
Median (range)	70.2 (40–87)	67.6 (34–87)
Sex		
Male	14 (27.5%)	24 (28.6%)
Female	37 (72.5%)	60 (71.4%)
MTX administration		2.5
Dose (mg/week), median (range)	7.26 (2.0–15.5)	5.84 (4.0–8.0)
Duration (month), median (range)	99.0 (1–360)	56.7 (2–193)
Autoimmune disease		
Rheumatoid arthritis	50 (98.0%)	36 (100%)
Others	1 (2.0%)	5 (SS, SLE, PMR)
Duration (month), median (range)	142.8 (6–396)	105.5 (27–360)
Primary site		
Gingiva	39 (76.5%)	
Tongue	6 (11.8%)	
Mouth floor	2 (3.9%)	
Plate	2 (3.9%)	
Others	2 (3.9%)	
Multiple sites		
(+)	11 (21.6%)	70 (83.3%)
Oral	5	
Others (lung and liver)	6	
(−)	40 (78.4%)	12 (14.3%)
EBV infection		
(+)	49 (96.1%)	25 (29.8%)
(−)	1 (2.0%)	51 (60.7%)
Unknown	1 (2.0%)	8 (9.5%)
Histopathological diagnosis		
DLBCL	38 (74.5%)	52 (61.9%)
Hodgkin's lymphoma	6 (11.8%)	14 (16.7%)
Others	7 (13.7%)	18 (21.5%)
Treatment		
W	39 (76.5%)	33 (39.3%)
C	5 (9.8%)	41 (48.8%)
W⟶C	4 (7.8%)	4 (4.8%)
Others	3 (5.9%)	6 (7.1%)
Recurrence		
(+)	2 (3.9%)	19 (22.6%)
(−)	40 (78.4%)	59 (70.2%)
Unknown	9 (17.6%)	6 (7.2%)

MTX, methotrexate; SS, Sjogren's syndrome; SLE, systemic lupus erythematosus; PMR, polymyalgia rheumatica EBV, Epstein-Barr virus; DLBCL, diffuse large B-cell lymphoma; W, withdrawal of MTX; C, chemotherapy
